# Association of body mass index with left ventricular diastolic dysfunction among ambulatory individuals with diabetes mellitus in rural Uganda: a cross-sectional study

**DOI:** 10.1186/s12872-022-02718-2

**Published:** 2022-06-20

**Authors:** Boniface Amanee Elias Lumori, Edwin Nuwagira, Fardous Charles Abeya, Abdirahman Ali Araye, Godfrey Masette, Charles K. Mondo, Samson Okello, Conrad Muzoora, Anthony Muyingo

**Affiliations:** 1 Department of Internal Medicine, Mbarara University of Science and Technology, P.O. Box 1410, Mbarara, Uganda; 2 Department of Microbiology and Immunology, Mbarara University of Science and Technology, Mbarara, Uganda; 3 Uganda Heart Institute, Makerere University College of Health Sciences, Kampala, Uganda; 4 Lown Scholars Program, Department of Global Health and Population, Harvard T.H. Chan School of Public Health, Boston, MA USA; 5 Division of Infectious Diseases and International Health, Department of Medicine, University of Virginia Health Systems, Charlottesville, VA USA

**Keywords:** Left ventricular diastolic dysfunction, Diabetes mellitus, Ambulatory individuals, Body mass index, Rural Uganda

## Abstract

**Background:**

Left ventricular diastolic dysfunction (LVDD) is a recognized complication of diabetes mellitus that precedes and is a risk factor for heart failure. We aimed to determine the prevalence of LVDD and its association with body mass index in ambulatory adults with diabetes mellitus in rural Uganda.

**Methods:**

We conducted a cross-sectional study, over 5 months, to enroll 195 ambulatory Ugandan adults living with diabetes mellitus for at least five years at Mbarara Regional Referral Hospital. We collected demographic, and clinical data and measured body mass index (BMI). Echocardiography was performed to determine LVDD by assessing the mitral inflow ventricular filling velocities (E/A and E/è ratios), tricuspid regurgitant jet peak velocity, and left atrium maximum volume index. We used logistic regression to estimate the odds ratio for the association of LVDD with BMI and evaluated the variation of associations by age and hypertension status.

**Results:**

Of the 195 participants, 141 (72.31%) were female, the mean age was 62 [standard deviation, 11.50] years, and the median duration of diabetes diagnosis was 10 [interquartile range, 7, 15] years. Eighty-six percent (n = 168) had LVDD with the majority (n = 127, 65.1%) of participants in the grade 1 category of LVDD. In the adjusted model, the odds of LVDD for each 1 kg/m^2^ increase in BMI was 1.11 [95% confidence interval 1.00, 1.25, p = 0.04]. The adjusted odds of LVDD among individuals aged ≥ 50 years with BMI ≥ 25 kg/m^2^ was 13.82 times the odds of LVDD in individuals aged < 50 years with BMI < 25 kg/m^2^.

**Conclusion:**

LVDD is prevalent and positively associated with BMI among ambulatory Ugandan adults living with diabetes mellitus for at least five years. The association was higher for older overweight/obese than younger individuals with normal weight. Future studies should focus on the effect of weight loss on LVDD as a possible target for the prevention of heart failure.

## Background

Despite advances in preventive and therapeutic care, individuals with diabetes mellitus (diabetes) have up to an eightfold risk of cardiovascular disease (CVD) compared to individuals without diabetes [1]. The overall risk of congestive heart failure is as high as 23%, which is three times the overall risk of congestive heart failure among individuals without diabetes [2]. Moreover, at least two-thirds of overall mortality in people with diabetes is due to CVD [1]. The morbidity and mortality due to diabetes are worse in Africa where diabetes and CVD often go undiagnosed until disease complications are evident [3, 4].

Aside from atherosclerotic and coronary diseases, several pathways including left ventricular diastolic dysfunction (LVDD) play a key role in the pathogenesis of heart failure and contribute to the heightened mortality [1, 5, 6]. LVDD per se is associated with an increased risk of subclinical atherosclerosis and ischemic heart disease [7]. In individuals with diabetes, the etiology of LVDD is often multifactorial with chronic hyperglycemia, overweight/obesity, and hypertension as the main contributing factors that eventually lead to morphological and physiological changes in the heart muscle [5, 8]. These factors synergistically worsen LVDD, and coronary blood flow thereby exacerbating ischemic heart disease and subsequently leading to overt heart failure [6, 9].

Body mass index (BMI) is a modifiable risk factor that could be a potentially interventional target to improve left ventricular diastolic function in individuals with diabetes. Interestingly, obese individuals with diabetes have repeated left ventricular myocardial damage possibly from complex pathophysiological interactions of obese-induced adipokines, leptin, and insulin [10] resulting in higher LVDD in obese individuals with diabetes compared to normal-weight individuals with diabetes. Also, obesity per se increases the risk of type 2 diabetes, hyperlipidemia, and hypertension which are known risk factors for LVDD [8, 11].

Despite the increase in the burden of overweight/obesity and diabetes in developing countries [12, 13], little is known about the association of overweight/obesity with the LVDD in individuals living with diabetes. Filling this knowledge gap could highlight the burden of LVDD in this high-risk population and identify groups with the highest-burden in whom targeted interventions are of maximum public health benefit.

Although echocardiographic screening for individuals with diabetes enables early detection of LVDD before overt heart failure sets in, in poor-resource settings screening echocardiography is either not available or affordable to those who need it. This represents a missed opportunity for optimization of treatment to prevent/delay disease progression and early diagnosis of heart failure [14]. In this study, we aimed to determine the prevalence of LVDD and its association with BMI among ambulatory adults with diabetes in Uganda.

## Methods

### Study design and setting

This was a single-center cross-sectional study of ambulatory adult individuals with diabetes at Mbarara Regional Referral Hospital (MRRH), the largest (more than 1500 active individuals) referral hospital in south-western Uganda.

### Participants’ recruitment and eligibility criteria

Between November 2017 and March 2018, we enrolled a consecutive sample of ambulatory individuals aged 18 years or greater with a self-reported history of diabetes and taking medications for glycemic control for 5 years or more. We excluded individuals with 1) a documented medical history of heart failure, valvular heart disease, arrhythmia, or thyroid disorders, and 2) a history of hazardous intake of alcohol (based on the Alcohol Use Disorder Identification Test). All individuals were consecutively identified and enrolled by a research assistant as they were logging in during their routine clinic visits. All consents used in this study were translated into the local language and informed written consent was obtained from all respondents.

### Data collection

We used a standardized quantitative questionnaire to collect data including participants’ age, occupational status, educational level, smoking habit, symptoms, human immunodeficiency virus (HIV) status, duration of diabetes, and history of hypertension.

#### Blood pressure

Blood pressure was measured following standardized procedures using automatic blood pressure monitors (Omron Corporation, Kyoto, Japan) with cuff sizes appropriate to individual participants. The participant was seated in a chair and allowed to rest for 5 min before three measurements were performed at 3-min intervals. We used the average of the second and third measurements to determine the blood pressure of each participant [15].

#### Body mass index

We measured weight and height using a weighing scale with a stadiometer (ADE Germany GmbH & Co. KG, Hamburg, Germany). Height was measured to the nearest 0.1 cm and weight was measured to the nearest kilogram. The plausible ranges for the anthropometric measurements were set as 100 to 200 cm for height, and 30 to 150 kg for weight. Values outside of these ranges were set to missing. We used height and weight to calculate BMI as weight (in kilograms) divided by the square of height (in meters), and categorized BMI as underweight (< 18.5 kg/m^2^), normal weight (18.5 to 24.9 kg/m^2^), overweight (25 to 29.9 kg/m^2^), or obese (> 30 kg/m^2^) [16].

#### Laboratory-based measurements

We collected blood specimens and measured glycated hemoglobin (HbA1c) (Siemens *DCA Vantage*™ *analyzer,* Siemens Healthcare Diagnostics Ltd, Frimley, Camberley, UK), low-density lipoproteins (LDL) (Humastar200™, Human Diagnostics Worldwide, Wiesbaden, Germany) and estimated urinary albumin to creatinine ratio (UACR) using the (Humastar200™, Human Diagnostics Worldwide, Wiesbaden, Germany). Laboratory tests were performed at the MRRH laboratory which has standardized internal quality control protocols and participates in external quality control programs by the National Health Laboratory Service.

#### Left ventricular diastolic dysfunction

F.C.A, a trained physician echocardiographer performed echocardiography (Philips HD7 XE Diagnostic ultrasound system, China) according to the recommendations of the American Society of Echocardiography and the European Association of cardiovascular imaging 2016 guidelines for the evaluation of LVDD [17]. We classed LVDD as (i) grade 1 if a mitral inflow E/A ratio ≤ 0.8 with peak E velocity ≤ 50 cm/second [or E/A ≤ 0.8 and peak E > 50 cm/second or E/A > 0.8 and < 2 in addition to either tricuspid regurgitation (TR) jet peak velocity > 2.8 m/second, average E/è > 14 (or lateral E/è ratio > 13 or septal E/è > 15) or left atrium (LA) maximum volume index > 34 ml/m^2^]; (ii) grade 2 if E/A ratio > 0.8 and < 2 (or ≤ 0.8 and peak E velocity > 50 cm/second) with at least two of the following parameters present, TR jet peak velocity > 2.8 m/second, average E/è > 14, or LA maximum volume index > 34 ml/m^2^; and (iii) grade 3 if E/A ratio ≥ 2 [17].

### Statistical analysis

After excluding poor quality echocardiographic imagines, we summarized normally distributed continuous variables by means (standard deviations) and the skewed continuous variables by medians (interquartile range). Categorical variables were grouped by frequencies and percentages. The prevalence of LVDD was determined as the proportion of those with LVDD in the overall study sample and further categorized by LVDD grades.

We used logistic regression to determine the odds ratio for the association between LVDD and BMI adjusted for confounding by a priori known factors including age (< 50 vs ≥ 50) years, sex (male vs female), hypertension (yes or no), HIV status (HIV-infected or HIV-uninfected), smoking (smoker or never), duration of diabetes (continuous) in years, HbA1c% (continuous), and LDL (continuous) mmol/L. In addition, we evaluated the variation of association of LVDD and BMI (< 25 vs ≥ 25 kg/m^2^) across; 1) age (< 50 vs ≥ 50) years and 2) hypertension (yes vs no) in the log odds (multiplicative) scale. All statistical analysis was conducted using Stata 13 (StataCorp, College Station, Texas, USA).

## Results

Overall, 998 individuals with diabetes were screened for eligibility. Of these, 782 had diabetes for less than five years, five had atrial fibrillation, four had a hazardous intake of alcohol, and one with history of hyperthyroidism. In addition, nine declined to consent, and two had poor echocardiographic windows. Of the 195 included in the analysis, the mean age was 62 [standard deviation (SD), 11.50] years and 141 (72.31%) were females. The median duration of diabetes was 10 [interquartile range (IQR) 7, 15] years with a median (IQR) HbA1c of 9.1% (7.7, 10.9). One hundred forty-six (74.87%) had a history of hypertension, median (IQR) blood pressure was 145 (128, 158) mmHg for systolic blood pressure (SBP) and 81 (75, 89) mmHg for diastolic pressure, 70 (36.08%) were overweight (BMI, 25–29.9 kg/m^2^) and 60 (30.93%) were obese (BMI, ≥ 30 kg/m^2^) (Table 1).

**Table 1:** Baseline socio-demographic and clinical characteristics

Characteristic	N = 195
Age (years) mean (*SD)	62 (11.50)
Female gender, n (%)	141 (72.31)
Level of education, n (%):	
None	47 (24.10)
Primary	91 (46.67)
Secondary	31 (15.90)
Tertiary	26 (13.33)
Occupation, n (%):	
Peasant farming	108 (55.38)
Business	19 (9.74)
Employed	30 (15.38)
Others	38 (19.47)
Duration of diabetes (years), median (*IQR)	10 (7, 15)
History of hypertension, n (%)	146 (74.87)
History of smoking, n (%):	
Never	160 (82.05)
Current	1 (0.51)
Former	34 (17.44)
Symptoms at enrolment, n (%):	
Paresthesia	119 (61.03)
Blurry vision	125(64.10)
Erectile dysfunction	15 (7.69)
Others	49 (64.10)
Systolic blood pressure at enrolment (mmHg), median (IQR)	145 (128, 158)
Diastolic blood pressure at enrolment (mmHg), median (IQR)	81 (75, 89)
Body mass index categories in kg/m^2^, n (%):	
Underweight (below 18.5)	5 (2.58)
Normal (18.5–24.9)	59 (30.41)
Overweight (25–29.9)	70 (36.08)
Obesity (30 and above)	60 (30.93)
Positive *HIV status, n (%)	14 (7.18)
Glycated hemoglobin (%), median (IQR)	9.1 (7.7, 10.9)
Glycated hemoglobin categories in %, n (%):	
Less than 7	27 (13.85)
7 and more	168 (86.15)
Low-density lipoproteins (mmol/L), median (IQR)	3.02 (2.3, 3.9)
Low-density lipoproteins categories (mmol/L), n (%):	
Optimal (< 2.6)	71 (36.41)
Near-optimal/above optimal (2.6–3.3)	39 (20.00)
Borderline high (3.4–4.1)	50 (25.64)
High (4.2–4.9)	18 (9.23)
Very high (≥ 5)	17 (8.72)
Urine albumin creatinine ratio (mg/mol), median (IQR)	266.6 (100, 500)
Left ventricular diastolic function categories, n (%):	
Normal	27 (13.85)
Grade 1 diastolic dysfunction	127 (65.13)
Grade 2 diastolic dysfunction	31 (15.90)
Grade 3 diastolic dysfunction	10 (5.13)

*SD* standard deviation, *IQR* interquartile range, *HIV* human immunodeficiency virus

**Fig. 1:**
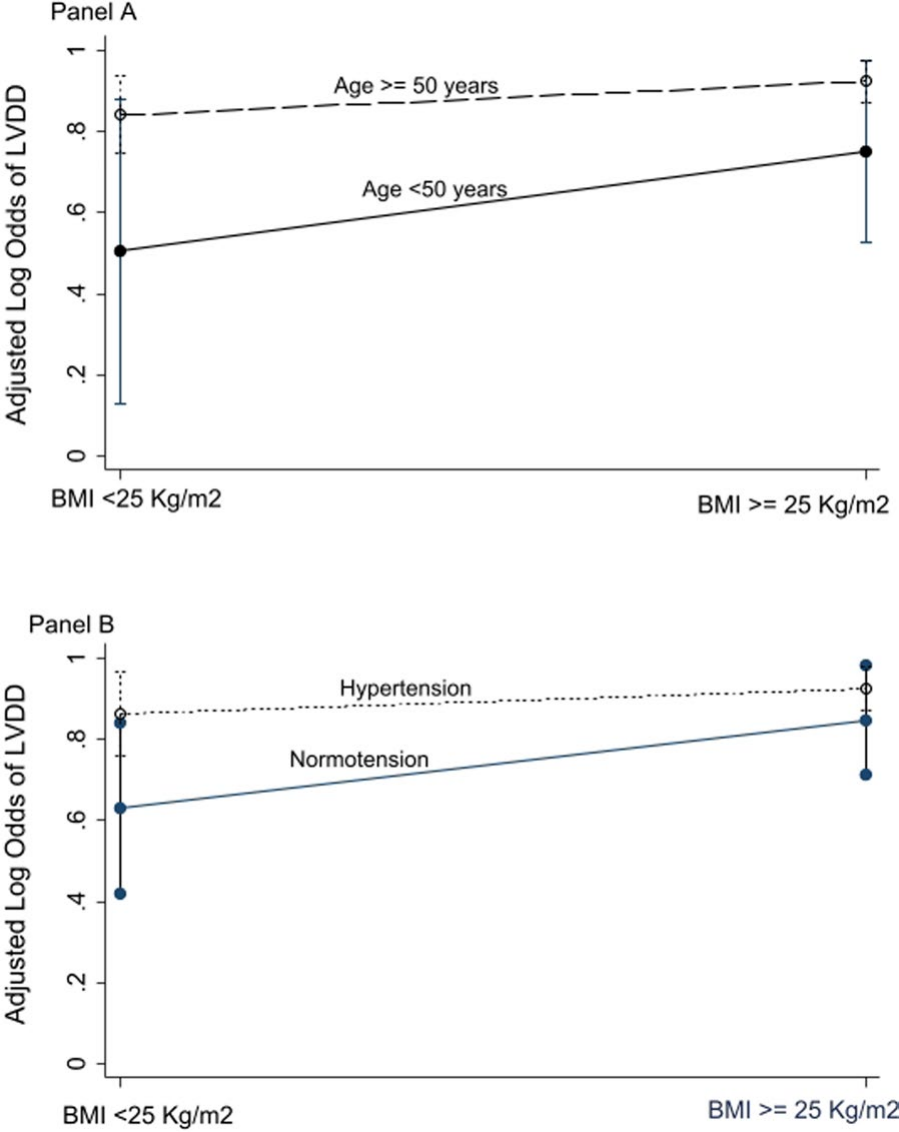
Variation of the association of left ventricular diastolic dysfunction (LVDD) and body mass index (BMI) across age (**A**) and hypertension status (**B**)

The prevalence of LVDD was found at 86.15% [95% confidence interval (CI) 0.80, 0.90]. Overall, 127 (65.13%) had grade 1 diastolic dysfunction (Table 1). In the fully adjusted model, we found that each 1 kg/m^2^ increase in BMI was associated with a 1.119 [95% CI 1.00, 1.24, p = 0.041] odds of LVDD (Table 2). In addition, we found that participants who were doubly exposed to BMI ≥ 25 kg/m^2^ and aged ≥ 50 years had 13.82 times higher odds of LVDD than those with BMI < 25 kg/m^2^ and aged < 50 years (Fig. 1A). The association was higher for those with obesity/overweight and hypertension compared to individuals with normal blood pressure and normal weight (Fig. 1B).

**Table 2:** Factors associated with increased odds of left ventricular diastolic dysfunction, n = 195

Variable	*OR (95%*Cl)	*P* value	*AOR (95%CI)	*P* value
Age ≥ 50 years	5.02 (1.84, 13.66)	0.002	4.25 (1.34, 13.47)	0.014
Female gender	1.12 (0.46, 2.73)	0.809	1.18 (0.42, 3.31)	0.749
Duration of diabetes (each year increase)	1.03 (0.96, 1.10)	0.416	0.99 (0.91, 1.07)	0.722
History of hypertension	4.09 (1.76, 9.50)	0.001	3.05 (1.15, 8.05)	0.025
History of *smoking	1.30 (0.419, 4.03)	0.648	1.01 (0.27, 3.84)	0.983
Body mass index (each kg/m^2^ increase)	1.11 (1.013, 1.21)	0.025	1.12 (1.00, 1.25)	0.041
Positive HIV status	2.18 (0.27, 17.38)	0.462	2.92 (0.31, 27.84)	0.351
Glycated hemoglobin (each % increase)	0.98 (0.84, 1.16)	0.853	1.04 (0.87, 1.24)	0.668
Low-density lipoproteins (each mmol/L increase)	1.00 (0.99, 1.00)	0.408	0.99 (0.98, 1.00)	0.161

*AOR* adjusted odds ratio, *OR* odds ratio, *CI* confidence interval, *Smoking* current and former smokers

## Discussion

In this study, the prevalence of LVDD was very high among ambulatory adults with diabetes in rural Uganda and highest among those older with overweight/obesity than those younger with normal weight; and among those overweight/obese with hypertension than those with normal weight and blood pressure.

In the current study, the prevalence of LVDD was higher than reported by prior studies. Muddu et al. reported the prevalence of LVDD as 55% in ambulatory adults with diabetes in Kampala, Uganda [18] while in Egypt [19] the prevalence of LVDD in individuals with diabetes was 61%. Both studies recruited newly diagnosed individuals with diabetes whereas the current study enrolled individuals with at least 5 years of living with diabetes. The heterogeneity could be because individuals with a longer duration of diabetes have prolonged hyperglycemia, prevalent hypertension, overweight/obesity, and dyslipidemia amongst other comorbid conditions which are known to cause LVDD [5, 8, 20]. In contrast, a study from Nigeria reported a 79% prevalence of LVDD [21], which is slightly lower than the findings of the current study. The study sample in the current study was on average 12 years older than the sample in the Nigerian study which in part explains the higher prevalence of LVDD since older age is associated with physiological delay in relaxation of the heart muscle causing diastolic dysfunction [22, 23].

Also, a study from Iraq reported a 62% prevalence of LVDD in asymptomatic adults with diabetes in an urban hospital [24]. While in another study from India, a 54% prevalence of LVDD among asymptomatic individuals with at least 5 years of type 2 diabetes was reported [9]. Both studies had younger populations compared to those in the current study. And in the latter study, individuals with hypertension were excluded. Another large population-based cross-sectional study from Australia reported a 34.7% prevalence of LVDD in a study population with a mean age of 69 years. Despite some similarities, the current study was hospital-based where individuals with the complicated forms of the disease are more likely to present and be enrolled than in the community-based studies [25]. Moreover, in Europe, a study done by Jorgensen and colleagues found a 19% prevalence of LVDD among individuals with type 2 diabetes receiving care in the specialized diabetic centers of an urban hospital [26], which is a much lower prevalence than in the current study. The difference could be explained by the fact that the European study population was recruited from highly specialized centers for diabetic care with proper management, multidisciplinary care, and follow-up, hence fewer diabetic-related cardiac complications [27].

Like the current study, previous studies reported increases in BMI to be associated with LVDD [19, 26, 28, 29]. Overweight and obesity are associated with increased plasma membrane content of fatty acids transporters such as plasma membrane-associated fatty acid-binding protein and fatty acid translocase which increases fatty acids uptake and utilization leading to cardiac steatosis [30] which induces overproduction of reactive oxygen species and ceramide causing myocardial damage [5, 31]. In addition, impairment of myocardial contractility can occur due to activation of adenosine triphosphate-dependent potassium channels leading to shortening of the action potential [5, 8, 32]. This myocardial damage is linked to the high release of cytokines from the cardiac adipocytes, enhanced by high insulin and leptin levels [10]. These provide a great opportunity to study weight reduction interventions and the use of lipid-lowering agents in individuals with diabetes who are overweight and have LVDD. Nevertheless, Muddu et al. found no association between overweight/obesity and diabetic cardiomyopathy [18]. Taken together, hypertension and increasing age among individuals with diabetes and obesity are independently associated with LVDD [33, 34]. Overweight/obesity also directly increases the risk of type 2 diabetes, hyperlipidemia, and hypertension which are primary risk factors for LVDD [8, 11].

Prior studies in Africa, Asia, and Europe similarly found advanced age was associated with LVDD [9, 18, 24, 26, 28]. This consistency could be explained by the physiological delay in relaxation of the left ventricle wall which occurs with increasing age hence abnormal diastolic function [22, 23]. However, Hassan et al., in a study in Egypt reported a null association between age and LVDD [19]. This contradicts the current study and could partially be explained by their exclusion of individuals older than 60 years and/or those with hypertension [19].

Unsurprisingly, hypertension was found to modify the association between BMI and LVDD in the current study. Several studies have reported hypertension as one of the major causes of left ventricular hypertrophy and diastolic dysfunction [28, 35–37]. Hypertension directly triggers the remodeling of cardiac walls through increases in the cardiac afterload and indirectly through the heightened activity of the renin-angiotensin system [38]. Individuals with diabetes and hypertension have higher left ventricular filling pressures which result in synergistic disastrous effects on cardiac myocytes than with either condition alone [39]. On contrary, Muddu and his colleagues didn’t find any association between hypertension and LVDD in their study [18]. Jain et al. described the associations of hypertension with LVDD to be amplified by diabetes and BMI of 30–35 kg/m^2^ [34].

Our study has several strengths. Though echocardiography has excellent sensitivity and specificity for the assessment of LVDD [40], it is invaluable in identifying additional findings associated with LVDD such as hypertrophic cardiomyopathy, primary valvular heart disease, and non-group 2 pulmonary hypertension, cardiac amyloidosis, pericardial disease, and high-output failure. However, this does not exclude LVDD misclassification. In addition, we used a rigorous and standard BMI measurement protocol. However, our results should be interpreted with the limitation of potential unmeasured confounding.

## Conclusion

In this study, LVDD is prevalent and positively associated with BMI among ambulatory Ugandan adults living with diabetes mellitus for at least five years. The association was higher for older overweight/obese than younger individuals with normal weight. Future studies should focus on the effect of weight loss on LVDD as a possible target for the prevention of heart failure.

## Data Availability

The datasets used during this study are available from the corresponding authors on reasonable request.

## Abbreviations

AOR: Adjusted odds ratio
BMI: Body mass index
CI: Confidence interval
CVD: Cardiovascular disease
HbA1c: Glycated hemoglobin
IQR: Interquartile range
LA: Left atrium
LDL: Low-density lipoproteins
LVDD: Left ventricular diastolic dysfunction
MRRH: Mbarara Regional Referral Hospital
MUREC: Mbarara University of Science and Technology Research and Ethics Committee
SD: Standard deviation
SBP: Systolic blood pressure
TR: Tricuspid regurgitations
UACR: Urine albumin to creatinine ratio
OR: Odds ratio
